# Case of Hypertrophia Cordis; with Remarks

**Published:** 1827-06

**Authors:** William Milligan

**Affiliations:** Physician to the Middlesex Infirmary, and to the Royal Infirmary for Children.


*Case of Hypertrophia Cordis; with Remarks.* By WILLIAM MILLIGAN, M.D. Physician to the MIDDLESEX INFIRMARY, and to the ROYAL INFIRMARY FOR CHILDREN.

SARAH FARRINGTON, ætatis forty-four, a washer-woman, of a slight figure and delicate constitution; is married, and has had eleven children; was admitted under my care at the Middlesex Infirmary, on the 9th of March. She complained of palpitation of the heart, dyspnœa, slight cough, general debility, and constipated bowels. Countenance sallow; tongue white and moist; appetite bad; pulse eighty-two, full, strong, and regular; pulsation very visible in the neck, more particularly on the right side, having on a superficial view the appearance of aneurism of the right carotid, but, on more careful examination, the calibre of the artery does not appear at all enlarged. On placing the hand over the heart, a strong "continuous" pulsation may be felt; and, on application of the stethoscope between the cartilages of the fifth and seventh ribs on the left side, the contraction of the ventricles appears prolonged, or as it were doubled; the sound tumultuous, with a strong impulse. The contraction of the auricles very short, and little sonorous. Catamenia had ceased twelve months since, but now appear about once in three months, scanty, and attended with pain in the lumbar region.

She was attacked last October with an acute pain in the region of the heart, attended with dyspnœa and cough, for which she was bled by a medical gentleman, and took some medicines, which relieved her for a time. For the last three months she has observed a palpitation in her left side, increased by severe exercise, going up stairs, or any agitation of mind. Attributes her disease to great care and anxiety, brought on by the misconduct and ill-treatment of her husband.

Aperiatur vena brachialis, et mittantur sanguinis unciæ octo.—℞. Pil. Rhæi compos. semidrachmam, divide in pilulas sex æq. capiat duas horâ somni, pro re nata, ad alvi solutionem.—℞. Infusi Quassiæ ℥j.; Tincturæ Digitalis m. xv. M. fiat haustus, ter die sumendus.—Ordered a spare vegetable diet, and to avoid exercise.

The medicines were continued until the 9th of April, with little relief to the symptoms, except the bowels being kept open. I now requested Dr. JAMES JOHNSON, the consulting physician to the Middlesex Infirmary, to meet me on that day. After a careful examination with the stethoscope, he was decidedly of opinion with myself that it was a case of hypertrophy of both ventricles of the heart. The following sedatives were then added to the other remedies:—

℞. Camphoræ (Sp. Vin. sol.) gr. tria; Extracti Hyoscyami gr. duo; Mucil. Acaciæ q. s. ut fiant pilulæ duæ æquales, habeat xij. tales. Capiat duas omni nocte.—Appr Emplast. Antim. Tartar. lateri dextro.

Hypertrophia Cordis consists of an increased thickness of the walls of the ventricles, of the septa and columnæ carneæ. The cavity of the heart is rather diminished; the blood is consequently sent into the arteries with greater force, which will account for the pulsations in the neck, as well as for the pulse at the wrist being full and strong. The dyspnœa and cough may be explained from the perpetual irritation the lungs suffer from the increased size of the heart, as well as the difficulty with which the blood is transmitted through its cavities. The palpitations may be referred to that law by which every irregular action of a muscular part, from whatever cause it arises, puts on the form of spasm or convulsion. The absence of the livid countenance and œdema of the extremities, point out the disease to be of short standing.

The only disease likely to be confounded with hypertrophy is "simple aneurism," or the "passive aneurism" of Corvisart. In the latter, the power by which the blood is propelled is rather diminished than increased; the cavity of the heart becomes more or less dilated, and the pulse at the wrists is much smaller, softer, and weaker, than usual. However, the pulse is "not always" soft and weak. Portal mentions one case in which the pulse was strong and hard until death. On examination with the stethoscope, the pulsations are clearer than natural, the ventricular contraction is distinct and sonorous; the extent in which we can hear the pulsation is increased, whilst the impulse is trifling. The more clear and louder the sound is, the greater the dilatation may be considered to be.

Angina Pectoris is known by the anxiety, difficulty of breathing, and pain of the arms, coming on by accessions, and much increased by severe exercise.

Aneurism of the aorta, or of the carotid arteries, can scarcely be mistaken for hypertrophia cordis: the former, however, may be a consequence of, and dependent upon, the latter.

The principal object kept in view in the treatment of this case, has been to render the circulation of the blood slower and weaker, by which means we certainly prevent a rapid increase of the disease, if we cannot cure it.

This case is related with the view of pointing out the symptoms by which the disease may be known to the junior members of the profession, and not of suggesting a remedy; which, I fear, would be no easy task. The recital, however, may serve to prevent fruitless attempts, and perhaps the aggravation of the symptoms, and consequent distress of the patient, where, upon due knowledge of the disease, art has evidently little to offer.

Should any further opportunity of illustrating this case be afforded me, I shall have much pleasure in stating it to the profession.

*Portman-street; April*, 1827.

